# Screening and validation of the optimal panel of reference genes in colonic epithelium and relative cancer cell lines

**DOI:** 10.1038/s41598-023-45174-4

**Published:** 2023-10-18

**Authors:** Yang Hu, Qi Jiang, Xiang Zhai, Liang Liu, Yuntian Hong

**Affiliations:** 1 Department of Gastroenterology, The First People’s Hospital of Jiande, Hangzhou, 311600 China; 2 Department of Biological Repositories, Zhongnan Hospital of Wuhan University, Wuhan, 430071 China; 3 Key Laboratory of Intestinal and Colorectal Diseases of Hubei Province, Zhongnan Hospital of Wuhan University, Wuhan, 430071 China; 4 Department of Orthopedic Surgery, Zhongnan Hospital of Wuhan University, Wuhan, 430071 China; 5 Department of Gastroenterology, Zhongnan Hospital of Wuhan University, Wuhan, 430071 China

**Keywords:** Reverse transcription polymerase chain reaction, Gastrointestinal cancer

## Abstract

Real-time quantitative polymerase chain reaction (RT-qPCR) is the most common method to determine mRNA expression, and Minimum Information for Publication of RT-qPCR Experiments (MIQE) proposes that a panel of reference genes for RT-qPCR is conducive to obtaining accurate results. This study aimed to screen and verify the optimal panel of reference genes in colorectal cancer (CRC) and normal colonic cell lines. In the study, eight candidate reference genes (GAPDH, ACTB, 18S, PPIA, B2M, SDHA, GUSB, and YWHAZ) were selected for RT-qPCR to detect their expression in NCM460, HT29, HCT116, SW480, SW620, DLD-1, LOVO and RKO cell lines. The stability of reference genes and the optimal panel were evaluated by geNorm, NormFinder, and BestKeeper software. As results, the expression levels of candidate reference genes differed in the colonic epithelial cell lines, and the number of optimal panel of reference genes is two. B2M and YWHAZ were the two most stable reference genes for NCM460, HCT116, SW620, LOVO, and RKO cell lines, while only one of B2M and YWHAZ was most stable in HT29 and SW480 cells. In DLD-1 cells, the stability of B2M and YWHAZ ranked 3rd and 6th, PPIA and GUSB were the most stable two. Furthermore, the YWHZA + B2M performed smaller intragroup differences than other panel or single reference gene. In conclusion, this study indicates the optimal panel of reference genes is YWHZA + B2M for the NCM460, HCT116, SW620, LOVO, RKO, SW480, and HT29 cell lines, but it is PPIA + GUSB in DLD-1 cell lines.

## Introduction

Real-time quantitative polymerase chain reaction (RT-qPCR) is currently frequently used for gene expression quantification, due to the advantage of rapidity, accuracy and sensibility^1–3^. A low number copies of target gene sequence are amplified based on the PCR technique which can’t be quantify by primary total RNA content^2^. Therefore, in order to obtain accuracy gene expression level of target gene, it’s important to use reference genes to normalize RT-qPCR data, which are supposed to stably express in cells or tissues under different experimental conditions^4–7^. Currently, housekeeping gene (HKG), including glyceraldehyde 3-phosphate dehydrogenase (GAPDH), beta actin (ACTB) as well as the 18S rRNA gene, are widely used for RT-qPCR as the reference genes^8–10^. However, it has been validated that HKG expression may be affected by different experimental conditions and pathological conditions such as bowel inflammation or cancer^6,11,12^. As a sensitive quantitative method, target gene expression may be incorrectly estimated as a result of variable reference genes. Therefore, screening and verifying reference genes that are stably expressed in different conditions is much more important.

Colorectal cancer (CRC) is the third most common cancer and the second cause of cancer death worldwide, RT-qPCR is widely used in the etiology, pathogenesis, pathological changes, and treatment of CRC^13,14^. However, it has been validated that ACTB and B2M in CRC can be affected by inflammation and be related to CRC stage, suggesting the expression of HKG are variable in CRC^11^. As a result, the differential gene expression of CRC will be wrongly estimated by RT-qPCR approach, owing to the use of reference genes that are not validated in CRC and relative cell lines. At present, there are several studies have aimed to demonstrate the best reference genes derived from CRC tissues and matched colonic epithelium. In a recent study held by the team of Malgorzata, 166 samples (35 CRC, 91 normal and 40 inflamed) were adopted and validated by geNorm and NormFinder, results showed that PPIA was the top-ranked reference gene for CRC tissue. And the pair RPLPO + PPIA was recommended as the best reference gene to normalize the RT-qPCR data for tissues from CRC and IBD. Moreover, ACTB, which is often used before was not recommended as a reference gene^11^. Other studies similar to Malgorzata, RPLPO, PPIA, HPRT1, and IPO8 are recommended as optimal reference genes^11,15–17^. However, there is no recommendation for commonly used in CRC research as candidate reference genes in different cell lines. Moreover, Minimum Information for Publication of RT-qPCR (MIQE) has shown that the use of a stable panel of reference genes, combination of more than 1 reference gene, is needed to obtain authentic results^18,19^, but there is no related report on human colonic epithelial and CRC cell lines.

In this study, the expression of eight candidate genes commonly used in CRC research (by consulting the literature) was assayed by RT-qPCR, and the stability of candidate genes and the optimal panel of reference genes were analyzed GeNorm, NormFinder and BestKeeper in seven CRC cell lines and one human normal colonic epithelial cell line. Furthermore, the reliability of the selected panel of reference genes was verified by normalizing the expression of APC, which is well known that the mRNA expression of APC is significantly decreased in HCT116 cells^20^. This study screened and validated the optimal panel of reference genes in the human normal colonic epithelial cell and CRC cell lines, which provides a methodological basis for studies related to CRC.

## Materials and methods

### Chemicals and reagents

TRIzol kit (CAS No. 5346994) was purchased from Invitrogen Co. (15596-026, Carlsbad, CA, USA); HiScript III RT SuperMix for qPCR (+ gDNA wiper) (CAS R323-01) and Taq Pro Universal SYBR qPCR Master Mix kits (CAS Q712-02) were purchased from Vazyme Biotech Co., Ltd. (Nanjing, China); All primers were designed and synthesized by Sangon Biotechnology Co., Ltd. (Shanghai, China). Isopropanol (CAS No. 67-63-0) and chloroform (CAS No. 67-66-3) were procured through Merck (Beijing, China). The other chemicals and agents were analytical grade.

### Cell culture

Human CRC cell lines HT29 (HTB-38), HCT116 (CCL-247EMT), SW480 (CCL-228), SW620 (CCL-227), DLD-1 (CCL-221), and LOVO (CCL-229) were purchased from ATCC; RKO was obtained from Procell (CL-0196, China); and NCM460 was provided by Cellverse (iCell-h373, China). HT29, HCT116, DLD-1, LOVO, and NCM460 were cultured in RPMI1640 (SH30809.01, HyClone, USA), and SW480, SW620, and RKO were grown in DMEM medium (C11995500BT, Gibco, USA). The medium was supplemented with 10% fetal bovine serum (FBS) (C0235, Gibco, AUS) and penicillin–streptomycin 1 × (C0222, Beyotime, China). All cells were maintained at 37 °C, 5% CO_2_ in a humidified atmosphere. Cells less than passage 10 were harvested at 90% confluence for further experiments.

### RNA extraction, integrity test and cDNA synthesis

The total RNA of cells was extracted using TRIzol Reagent (15596-026, Invitrogen, USA). In brief, the cells were washed with PBS, and 1 mL of trizol was added to the cell culture plastic. By pipetting 3–5 times, cell lysis was collected into 1.5 mL Centrifuge tubes (enzyme-free) and allowed to stand at room temperature for 10 min. Subsequently, 200 μL chloroform was added and mixed by a vortex. After placed for 5 min on ice, the samples were centrifuged in a centrifuge (Centrifuge 5430R, Eppendorf, Germany) at 12,000 rpm for 15 min at 4 °C. The supernatant (400 μL) of the upper layer was transferred into a new EP tube and blended with an equal volume of isopropyl alcohol, and then the mixture was allowed to precipitate at room temperature for 10 min. The samples were then centrifuged at 12,000 rpm for 10 min at 4 °C to precipitate the total RNA, which was washed with 1 mL of precooled 75% ethanol twice with a centrifuge at 9500 rpm for 5 min at 4 °C and dissolved with enzyme-free water. NanoDropOne (ThermoFisher Scientific, USA) was used to assay the RNA concentration and purity. It suggested the RNA purity was acceptable when A260/280 was between 1.8 and 2.1.

RNA integrity was assayed by agarose gel electrophoresis^21^. Briefly, 1% agarose gels containing 3 μL of 10 mg/ml ethidium bromide in each gel were used for most RNA integrity assessments. Marker (DL5000, Vazyme Biotech Co., Ltd., Nanjing, China) and 2 μL total RNA samples mixed with 2 × RNA loading buffer (R0215, Beyotime™, Shanghai, China) at a ratio of 1:1 were added to into separate wells of the gel. Electrophoresis was performed in TAE buffer at a condition of 100 voltage, and stopped at 2–3 cm from the edge of the gel. The gels were observed and photographed using a gel imaging system (JY04S-3C, Beijing JUNYI Electrophoresis Co., Ltd, China). 28S:18S band intensity ratios were calculated by Image J (n = 3 × 3), and ratios higher than 1:1 were considered eligible samples^22^. The eligible RNA samples were used to synthesize cDNA by HiScript III RT SuperMix for qPCR (+ gDNA wiper) kits (R323-01, Vazyme, China) following the manufacturer’s protocol. The genomic DNA was first removed by a mixture of 4 μL 4 × gDNA wiper Mix, 1 μg RNA and RNase-free ddH_2_O (added to 16 μL) and incubating at 42 °C for 2 min by a PCR instrument. Then, 4 μL 5 × HiScript II qRT SuperMix II was added for the reverse transcription step, which was performed at 50 °C for 15 min followed by 85 °C for 5 s. Finally, the samples were diluted with enzyme-free water at 1:5 and stored at − 20 °C for further analysis.

### Candidate reference genes, primers design, and RT-qPCR

As shown in Table S1, 8 reference genes (GAPDH, ACTB, 18S, PPIA, B2M, SDHA, GUSB, YWHAZ) commonly used in colorectal research were selected as candidate genes by consulting the literature. The complete cDNA sequence of the candidate genes was obtained from NCBI's Entrez Nucleotides database. Primer Premier 5.0 (PREMIER Biosoft International) was used for primer design, and then the designed primers were input into the BLAST database for homology comparison. Melting curve and standard curve were used to verify primer specificity and amplification efficiency. As previously described^23^, the standard curve reaction program is at 95 °C for 10 min, 95 °C for 15 s and 60 °C for 1 min for 40 cycles, and the melting curve is at 95 °C for 15 min, 60 °C for 30 s, and 95 °C for 15 min. After sample gradient dilution of 2^8^ times, the standard curve was analyzed by StepOnePlus™ Real-Time PCR System (AppliedBiosystems by ThermoFisher Scientific™, USA). The amplification efficiency (E) of primers was calculated by using the slope and R^2^ of the standard curve, which was acquired according to $E(\%)=({10}^{-\frac{1}{slope}}-1)\times 100$. The specific primer sequences were shown in Table S2.

The expression levels of candidate genes were detected by RT-qPCR in NCM460, HT29, HCT116, SW480, SW620, DLD-1, LOVO and RKO cell lines using StepOnePlus™ Real-Time PCR System according to the manufacturer's protocol of Taq Pro Universal SYBR qPCR Master Mix kits. The 10 μL reaction system, containing 0.2 μL forward and reversed primers, 1 μL cDNA, 3.6 μL ddH_2_O and 5 μL SYBR, was used in the RT-qPCR assay of this study. The amplification curve reaction program was at 50 °C for 2 min, 95 °C for 2 min, 95 °C for 15 s and 60 °C for 1 min for 40 cycles. The 2^−ΔΔCt^ mode in StepOnePlus™ Real-Time PCR System was used to obtain the Ct values of the candidate reference genes and the 2^−ΔΔCt^ method was used for the statistical analysis of APC gene expression differences in the validation experiments. A single reference gene or the geometric mean of screened reference gene panel was used to normalize APC expression levels^24^. Three technical repetitions were performed in this study.

### Software

GeNorm (version 3.5, http://medgen.ugent.be/~jvdesomp/genorm/)^25,26^, NormFinder (http://www.mdl.dk/publicati-onsnormfinder.htm)^26,27^, and BestKeeper (http://www.wzw.tum.de/gene-quantification/bestkeeper.html)^28^. are three commonly used reference gene stability analysis software based on statistics. GeNorm determines the stability of the reference gene by the average stability value of M. The smaller the M value is, the more stable the reference gene expression is. When M is less than 1.5, it indicates that the expression of the gene is stable, which can be used as a reference gene. In addition, geNorm can also calculate the optimal number of reference genes by judging the pairing coefficient of variation (the V values) of standardization factor after introducing a new reference gene. When Vn/(n + 1) < 0.15, the number is n^23^. BestKeeper directly analyzes the standard deviation (SD), correlation coefficient (r), and coefficient of variation (CV) of circulation threshold (Ct) value to judge the stability of gene expression; the smaller the SD and CV, the more stable the reference gene expression was; when the SD value is more than 1, it indicates that the gene expression is unstable and can’t be used as a reference gene^29–31^. The same as geNorm, normFinder obtains the average stable value (M) of gene expression by calculating the within-group and between-group variance of C_t_ value. The smaller the m value is, the more stable the gene expression is, but only one of the most stable reference genes can be screened^29–31^. In this study, the most stable reference genes in normal colonic epithelial cells and colonic cancer cell lines were analyzed by the above three software. The comprehensive ranking was obtained by calculating the geometric mean of each software ranking, and finally the optimal panel of reference gene in each cell line was screened.

### Statistical analysis

Data statistics and graphing were operated in Prism 8 (Graph Pad, USA). Quantitative data were reflected as the mean ± SEM. An unpaired two-tailed Student's t-test was used for statistical test. *P* < 0.05 was considered statistically significant.

## Results

### RNA integrity and the specificity and amplification efficiency of the candidate gene primers

**Figure 1:**
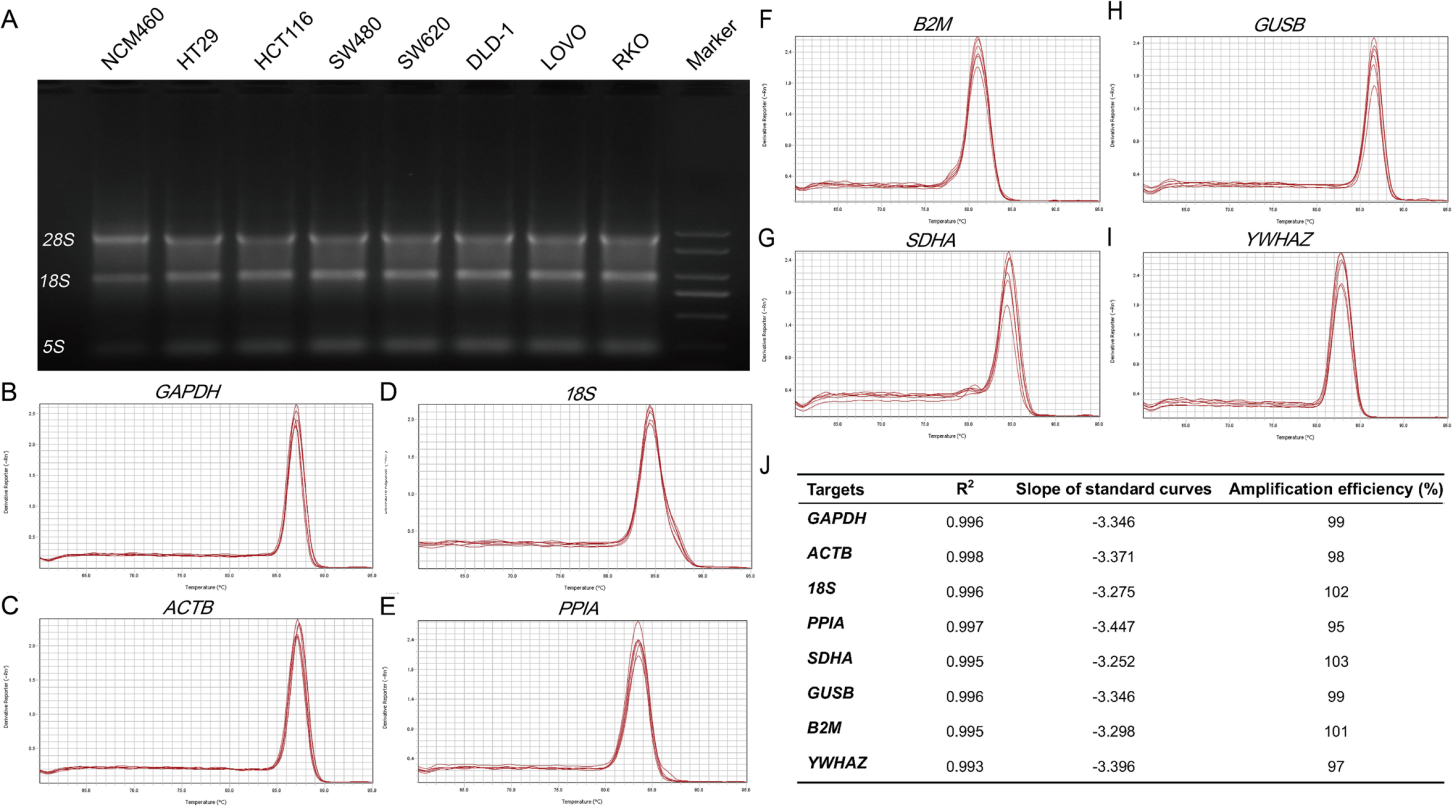
The integrity of total RNA and the specificity and amplification efficiency of the primers. (**A**) The cropped gels showing the total RNA integrity by RNA gel electrophoresis; The melt curves of GADPH (**B**), ACTB (**C**), 18S (**D**), PPIA (**E**), SDHA (**F**), GUSB (**G**), B2M (**H**) and YWHAZ (**I**); (**J**) The amplification efficiencies and standard curve parameters of the candidate reference genes.

The integrity of total RNA samples, the specificity and the amplification efficiency of PCR primers are important factors affecting the accuracy of RT-qPCR results. Therefore, the above factors are verified first. The agarose gel electrophoresis showed that the integrity of total RNA was eligible (Fig. 1A, S1). All melt curves showed a single peak (Fig. 1B–I), indicating all the selected reference genes primers showed a good specificity. The standard curves showed R^2^ ≥ 0.993, suggesting there was a good linear relationship between template cDNA and CT values (Fig. 1J). Moreover, The amplification efficiency of the primers calculated by slope and R^2^ showed that the values were 97–103%, which is in line with the RT-PCR principle of MIQE^32^.

### Basic expression levels of the candidate reference genes in different cells

**Figure 2:**
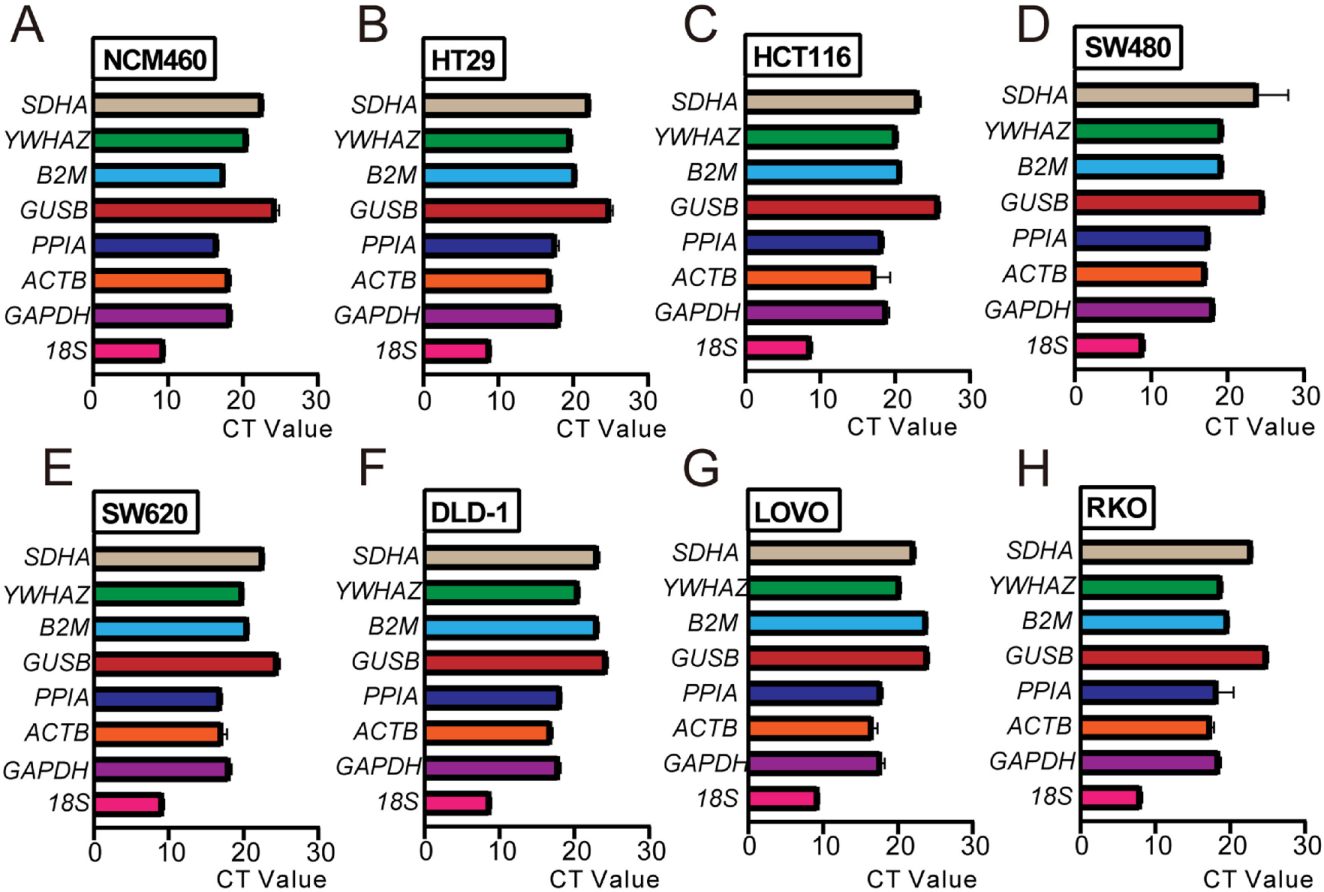
Average CT value of reference genes in different cells. RT-qPCR was acclimated to ascertain the expression of the candidate reference genes in NCM460 (**A**), HT29 (**B**), HCT116 (**C**), SW480 (**D**), SW620 (**E**), DLD-1 (**F**), LOVO (**G**) and RKO (**H**) cell lines.

The reference genes have stable expression, and the general level is high. Our results showed that the average Ct values of the candidate genes were between 8 and 27 in the human colonic epithelial cell (NCM460) and cancer cell lines (HT29, HCT116, SW480, SW620, DLD-1, LOVO and RKO), and the expression of 18S was the highest while GUSB was the lowest in all cells except for LOVO cell line (Fig. 2A–H). Further analysis showed that the expression levels of reference genes in different cells are inconsistent. For example, the expression of YWHAZ in NCM460 cells is lower than that in B2M, which is the opposite in HT29 cells (Fig. 2A, B), indicating the stability of reference gene expression may be changed in human normal colonic epithelial cells and cancer cells.

### The stability analysis of the candidate reference genes in colonic epithelial cells

**Figure 3:**
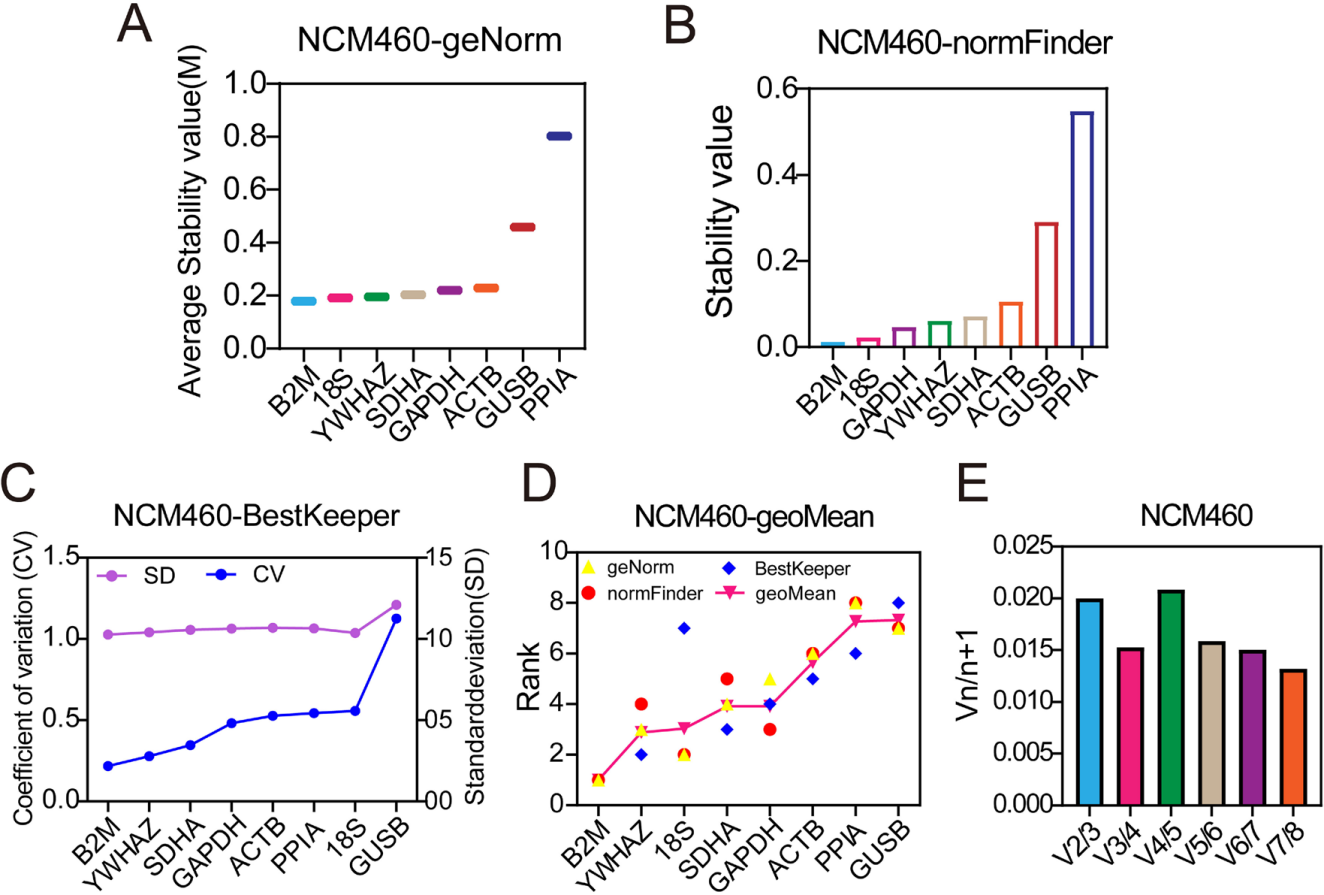
Stability of each reference gene in normal epithelial cells. The stability of the candidate reference genes was analyzed by geNorm (**A**), normFinder (**B**) and BestKeeper (**C**); (**D**) The comprehensive stability ranking of the candidate reference genes; (**E**) Paired coefficient of variation (CV) of standardization factor’s variation after introducing a new reference gene in NCM460 cells.

First, we analyzed the stability of reference genes in normal colonic epithelial cell lines (NCM460). GeNorm showed the stability of the candidate reference genes from high to low was B2M > 18S > YWHAZ > SDHA > GAPDH > ACTB > GUSB > PPIA (Fig. 3A); NormFinder showed the order was B2M > 18S > GAPDH > YWHZA > SDHA > ACTB > GUSB > PPIA (Fig. 3B); BestKeeper showed the order was B2M > YWHAZ > SDHA > GAPDH > ACTB > PPIA > 18S > GUSB (Fig. 3C).

We calculated the geometric mean of ranking in three software to further analyze the comprehensive rank of reference genes in normal colonic epithelial NCM460 cell line. The results showed the comprehensive ranking of the most to least stable genes in NCM460 cells were B2M > YWHAZ > 18S > SDHA > GAPDH > ACTB > PPIA > GUSB (Fig. 3D). Furthermore, the paired coefficients of standardization factor’s variation after introducing a new reference gene calculated by geNorm showed V2/3 were less than 0.15 (Fig. 3E), suggesting the best number of optimal reference genes is two. Therefore, B2M + YWHAZ was the optimal panel of reference gene in NCM460 cell line.

### The stability analysis of the candidate reference genes in colonic cancer cell lines

**Figure 4:**
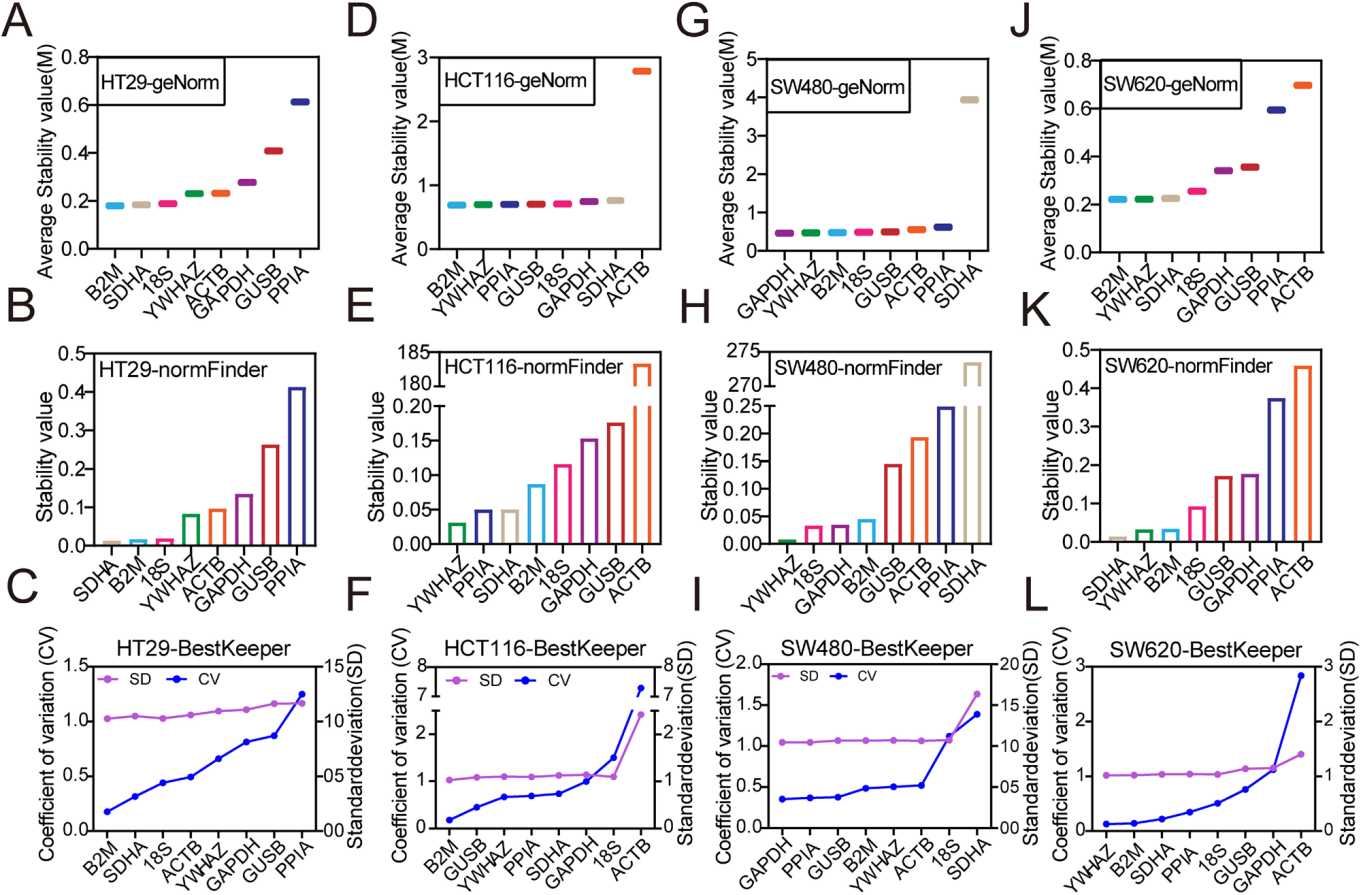
Stability of each reference gene in four colonic cancer cell lines. GeNorm, normFinder and BestKeeper analyzed the stability of the candidate reference genes in HT29 (**A**–**C**), HCT116 (**D**–**F**), SW480 (**G**–**I**), SW620 (**J**–**L**).

We further analyzed the stability of reference genes in colonic cancer cell lines (HT29, HCT116, SW480, SW620, DLD-1, LOVO and RKO). In HT29 cell line, geNorm showed the most to least stable genes were B2M > SDHA > 18S > YWHAZ > ACTB > GAPDH > GUSB > PPIA (Fig. 4A); normFinder showed the order was SDHA > B2M > 18S > YWHAZ > ACTB > GAPDH > GUSB > PPIA (Fig. 4B); BestKeeper showed the order was B2M > SDHA > 18S > ACTB > YWHAZ > GAPDH > GUSB > PPIA (Fig. 4C). In HCT116 cell line, geNorm showed the ranking of most to least stable genes were B2M > YWHAZ > PPIA > GUSB > 18S > GAPDH > SDHA > ACTB (Fig. 4D); normFinder showed the order was YWHAZ > PPIA > SDHA > B2M > 18S > GAPDH > GUSB > ACTB (Fig. 4E); BestKeeper showed the order was B2M > GUSB > YWHAZ > PPIA > SDHA > GAPDH > 18S > ACTB (Fig. 4F). In SW480 cell line, geNorm showed the ranking was GAPDH > YWHAZ > B2M > 18S > GUSB > ACTB > PPIA > SDHA (Fig. 4G); normFinder showed the order was YWHAZ > 18S > GAPDH > B2M > GUSB > ACTB > PPIA > SDHA (Fig. 4H); BestKeeper showed it was GAPDH > PPIA > GUSB > B2M > YWHAZ > ACTB > 18S > SDHA (Fig. 4I). In SW620 cell line, geNorm showed the ranking of most to least stable genes were B2M > YWHAZ > SDHA > 18S > GAPDH > GUSB > PPIA > ACTB (Fig. 4J); normFinder showed the ranking was SDHA > YWHAZ > B2M > 18S > GUSB > GAPDH > PPIA > ACTB (Fig. 4K); BestKeeper showed the ranking was YWHAZ > B2M > SDHA > PPIA > 18S > GUSB > GAPDH > ACTB (Fig. 4L).

**Figure 5:**
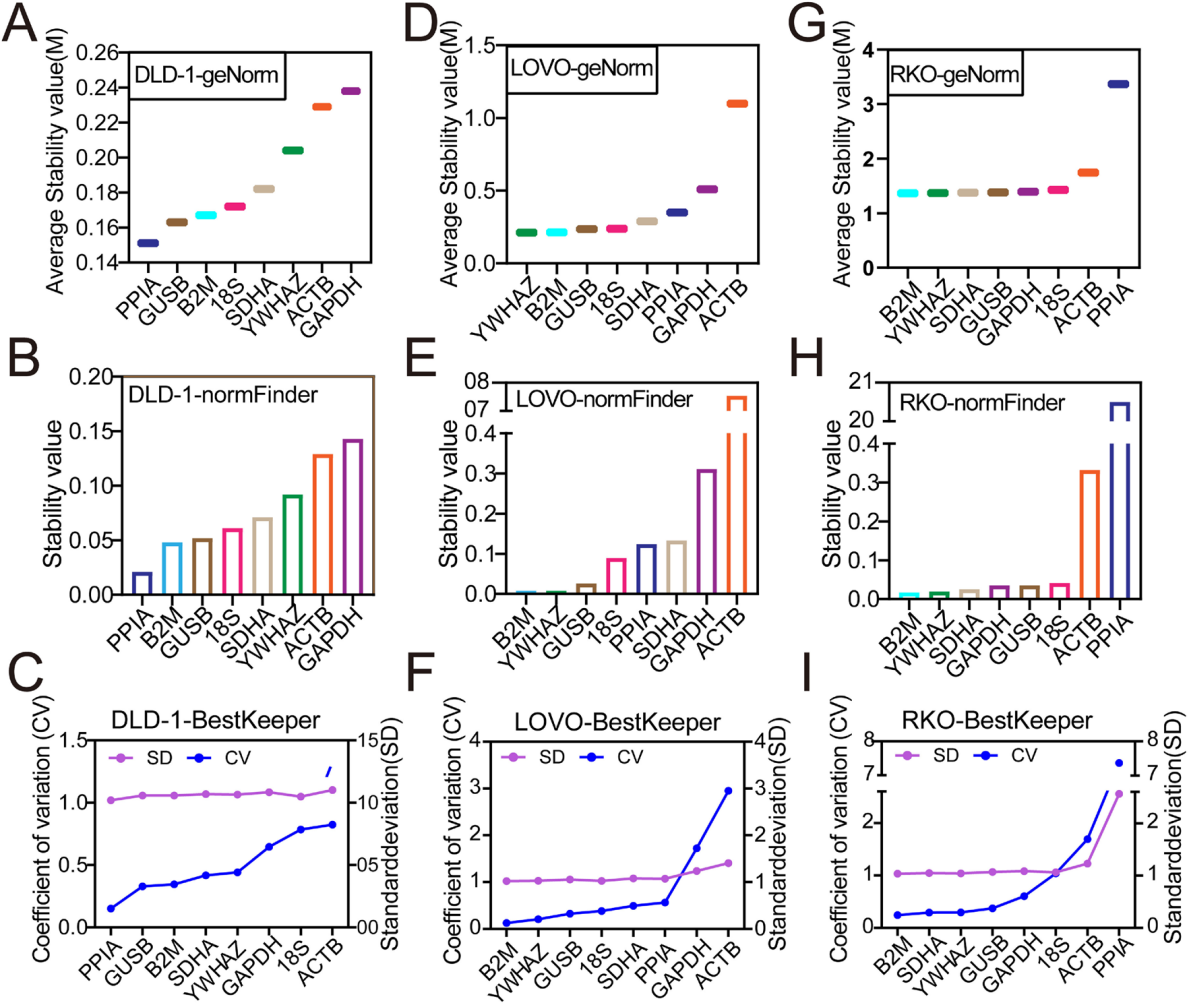
Stability of each reference gene in another three colonic cancer cell lines. GeNorm, normFinder and BestKeeper analyzed the stability of the candidate reference genes in DLD-1 (**A**–**C**), LOVO (**D**–**F**) and RKO (**G**–**I**).

In DLD-1 cell line, geNorm showed the ranking was PPIA > GUSB > B2M > 18S > SDHA > YWHAZ > ACTB > GAPDH (Fig. 5A); normFinder showed the ranking was PPIA > B2M > GUSB > 18S > SDHA > YWHAZ > ACTB > GAPDH (Fig. 5B); BestKeeper showed the ranking was PPIA > GUSB > B2M > SDHA > YWHAZ > GAPDH > 18S > ACTB (Fig. 5C). In LOVO cell line, geNorm showed the ranking was YWHAZ > B2M > GUSB > 18S > SDHA > PPIA > GAPDH > ACTB (Fig. 5D); normFinder showed the ranking was B2M > YWHAZ > GUSB > 18S > PPIA > SDHA > GAPDH > ACTB (Fig. 5E); BestKeeper showed it was B2M > YWHAZ > GUSB > 18S > SDHA > PPIA > GAPDH > ACTB (Fig. 5F). In RKO cell line, geNorm showed the ranking was B2M > YWHAZ > SDHA > GUSB > GAPDH > 18S > ACTB > PPIA (Fig. 5G); normFinder showed it was B2M > YWHAZ > SDHA > GAPDH > GUSB > 18S > ACTB > PPIA (Fig. 5H); BestKeeper showed it was B2M > SDHA > YWHAZ > GUSB > GAPDH > 18S > ACTB > PPIA (Fig. 5I).

Similarly, we further calculated the comprehensive ranking of the candidate reference genes. The ranking was shown in Table 1. The two most stable reference genes B2M and YWHAZ in normal colon epithelial cell lines were also the most stable in HCT116, SW620, LOVO and RKO cell lines, while only one of B2M and YWHAZ was most stable in HT29 and SW480 cells. In DLD-1 cell line, the stability of B2M and YWHAZ ranked 3rd and 6th respectively (Table 1). The same as NCM460, the paired coefficients of standardization factor’s variation after introducing a new reference gene V2/3 in all colonic cancer cell lines was less than 0.15 (Table S3), suggesting the number of optimal panel of reference gene was two. Due to the stability values M of YWHAZ and B2M were less than 1.5 and the stability ranked in the top four in HT29 and SW480 cell lines, B2M + YWHAZ can also be used as the optimal panel of reference gene in HT29, HCT116, SW480, SW620, LOVO and RKO cell line. But PPIA + GUSB was the optimal panel of reference gene in DLD-1 cell line.

**Table 1:** Comprehensive ranking of reference genes stability in colonic cancer cell lines.

Rank	HT29	HCT116	SW480	SW620	DLD-1	LOVO	RKO
1	*B2M*	*YWHAZ*	*YWHAZ*	*YWHAZ*	*PPIA*	*B2M*	*B2M*
2	*SDHA*	*B2M*	*GAPDH*	*B2M*	*GUSB*	*YWHAZ*	*YWHAZ*
3	*18S*	*PPIA*	*B2M*	*SDHA*	*B2M*	*GUSB*	*SDHA*
4	*YWHAZ*	*GUSB*	*18S*	*18S*	*18S*	*18S*	*GUSB*
5	*ACTB*	*SDHA*	*GUSB*	*PPIA*	*SDHA*	*SDHA*	*GAPDH*
6	*GAPDH*	*18S*	*PPIA*	*GUSB*	*YWHAZ*	*PPIA*	*18S*
7	*GUSB*	*GAPDH*	*ACTB*	*GAPDH*	*GAPDH*	*GAPDH*	*ACTB*
8	*PPIA*	*ACTB*	*SDHA*	*ACTB*	*ACTB*	*ACTB*	*PPIA*

### Verification of stability and accurateness of the selected reference genes

**Figure 6:**
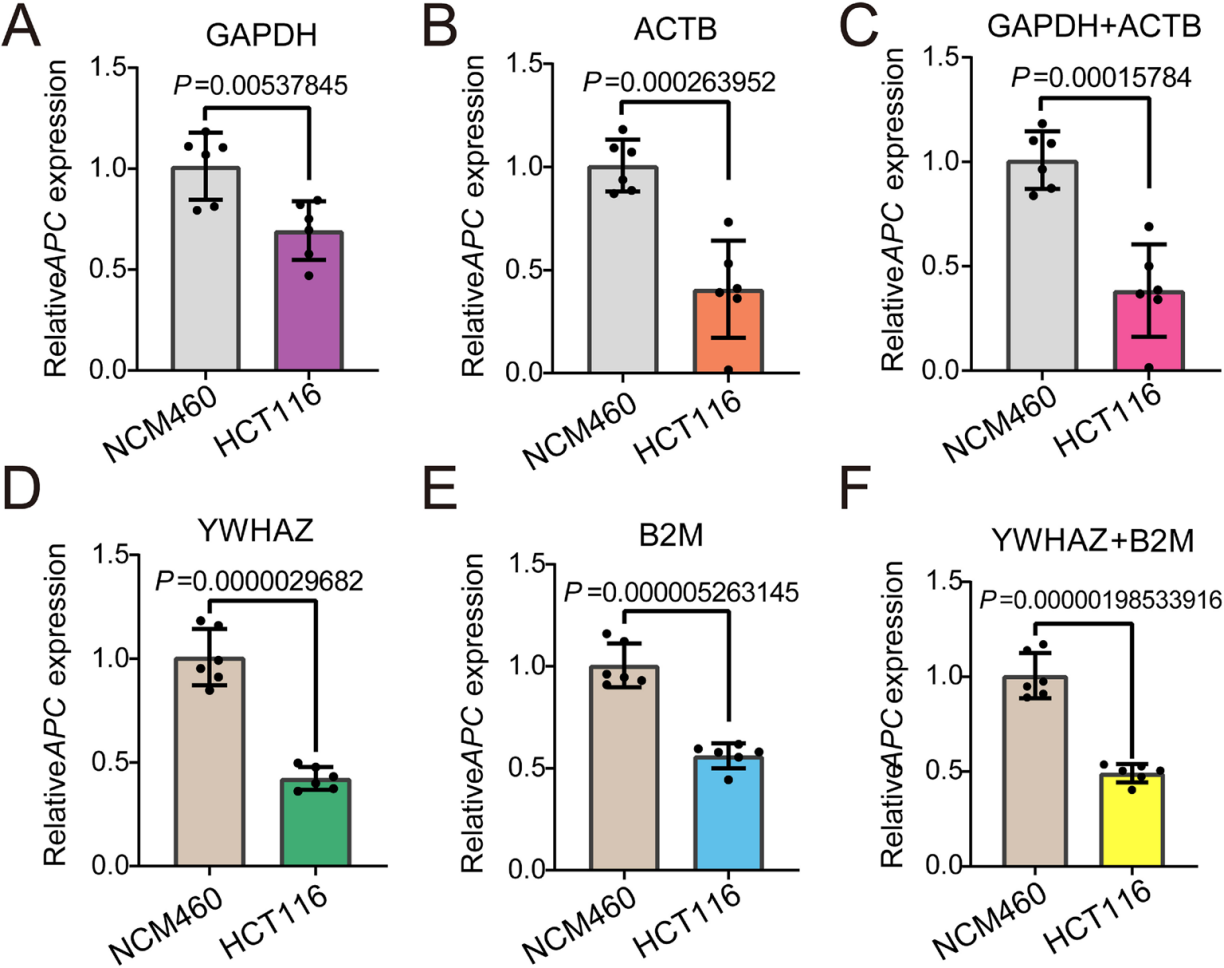
Different reference genes normalized APC mRNA expression in HCT116 cell. GADPH (**A**), ACTB (**B**), GAPDH + ACTB (**C**), YWHAZ (**D**), B2M (**E**) and YWHAZ + B2M (**F**), respectively, normalized APC mRNA expression. Datasets reflected means ± SD.

To further verify the stability and believability of the selected panel of reference gene, we selected HCT116 cell line for further experiment. APC is a well-known tumor suppressor gene. Various experiments have suggested that the mRNA expression of APC in HCT116 cells is significantly decreased^20^. First, we standardized APC expression with GAPDH and/or ACTB, and the results showed that there was a more significant statistical difference normalized with the panel of reference genes (Fig. 6A–C). We then normalized APC expression with the screened reference genes (B2M and YWHAZ). The results showed that, consistently, there was also a more significant statistical difference normalized with the panel of reference genes compared to a single reference gene (Fig. 6D–F). Moreover, whether screened single or panel of reference genes, the results indicated a more significant statistical difference normalized with B2M and YWHAZ compared to GAPDH and ACTB (Fig. 6A–F).

## Discussion

RT-qPCR is a robust tool for biomedical research, providing a sensitive and quantification method to determine gene expression. At present, geNorm, NormFinder, and BestKeeper are most common software used for gene expression stability analysis^29–31^. In this study, we use the three above software to estimate the stability of eight candidate reference genes in normal human colonic epithelial cell and seven CRC cell lines. Due to different algorithms in three tools, there was slight discrepancy in the stability ranking of candidate genes. For example, B2M was the best suitable reference gene in NCM460, HT29, HCT116, SW620, RKO cell lines according to geNorm. However, NormFinder showed that SDAH was the most stable reference gene for HT29 and SW620 cell lines, and B2M ranked second and third, respectively. YWHAZ ranked first in HCT116 and SW620 cell lines according to BestKeeper. PPIA was the best suitable reference for DLD-1 cell line according to three tools, but it was the lowest stable in all tools for RKO cell line. Therefore, comprehensive ranking was made, and YWHAZ and B2M were the two most stable reference genes in NCM460, HCT116, SW620, LOVO, and RKO cell lines, respectively, while only one of B2M and YWHAZ was the most stable in HT29 and SW480 cells. However, PPIA and GUSB were just the two most stable reference genes in DLD-1 cell line.

The optimal reference gene may change in different tissues, experiment designs, or diseases, even vary in cell lines and tissues of the same tumor^25,33,34^. Most recent research from the team of Sørby reported that PPIA was the most optimal reference gene to normalize gene expression in colon cancer even metastatic colon cancer^15^. In their research, PPIA was one of top two stable reference genes by using geNorm, and GUSB was ranked second by CtCV%, which was in concordance with our findings of DLD-1 cell line. However, our result showed that GUSB and PPIA was not the most stable reference gene when compared with other genes except in DLD-1 cell line. To our surprise, PPIA even was the lowest stable reference gene in some cell lines, such as RKO and HT29. Same as GUSB, in most cell lines, it was not also the most stable reference gene. This difference may be explained by different experiment designs. In Sørby’s research, they included tissues both cancer and matched normal mucosa from 18 non-metastatic colon cancer patients and 20 metastatic cancer patients, while we tested in diverse colon cancer cell lines. Furthermore, the percentage of cancer cells in the tissues they accepted was not assessed, indicating that total RNA was mixed with other RNA from colonic cells. Another study suggested that the panel of B2M was the most suitable reference genes to normalize RT-qPCR data in CRC tissue^35^. Identically, our result showed that B2M was also the most stable reference gene in majority cell lines, especially in NCM460, RKO, and LOVO. Although the study demonstrated that the expression of B2M gene was variable^15^, our result showed that B2M ranked top three in all cell lines, which further indicates that B2M is stably expressed in human colonic epithelial and colorectal cancer cell lines.

According to the Minimum Information for Publication of PCR Experiments (MIQE) guidelines in 2013 and updated in 2020^18,36^, it is suggested that the accuracy of the RT-qPCR results can be affected by using only one single reference gene to standardize the expression of genes. In addition, Vandesompele et al. found that the error of using a single reference gene was 6.5-fold compared with using multiple genes data for normalization^25^. Therefore, to ensure the believability of RT-qPCR, it is important to screen the stable panel of reference genes in human colonic epithelial and colorectal cancer cell lines. Studies suggested that when Vn/(n + 1) < 0.15 in geNorm, the number of optimal panel of reference genes is n^23^. Our result showed that V2/3 in all cell lines we selected was less than 0.15, suggesting that the number of optimal panel of reference genes was two. According to the comprehensive ranking, YWHZA + B2M was naturally the optimal panel of reference genes in NCM460, HCT116, SW620, LOVO, and RKO cell lines, while it was PPIA + GUSB in DLD-1 cell line. In addition, because the stability values M of YWHAZ and B2M were less than 1.5 and one of B2M and YWHAZ was most stable and the stability ranked in the top four in HT29 and SW480 cells, it is worth noting that, considering the experimental simplicity, repeatability and economy, YWHZA + B2M can be also used as the optimal panel of reference gene in HT29 and SW480 cell lines.

Previous studies showed that transcriptomic and proteomic expression levels of APC also significantly reduced in HCT116 cell lines^20^. Therefore, APC expression was normalized by different reference genes in HCT116 cell lines to further verify the believability of reference genes screened in this study. The results showed there was more significant statistical difference in APC expression normalized by the most stable reference screened in this study than the commonly used reference genes. Moreover, there was a more significant statistical difference in APC expression normalized by whether the commonly used panel of reference genes (GAPDH + ACTB) or the optimal panel of reference genes (YWHZA + B2M) screened in this study than that of a single reference gene among them. These results suggested that the selected the optimal panel of reference genes reduced the differences within the group and was more conducive to obtaining an authentic experimental conclusion.

## Conclusion

In this study, to identify the optimal panel of reference genes to normalize RT-qPCR data in CRC and colonic epithelial cell lines, we applied geNorm, NormFinder, and BestKeeper to estimate the stability of eight reference genes. Our finding showed that YWHAZ + B2M is the optimal panel of reference genes in the normal human colonic epithelial cell lines (NCM460) and some CRC cell lines (HCT116, SW620, LOVO, RKO, SW480, and HT29), while it is PPIA + GUSB in DLD-1 cell lines. This study provided a theoretical and experimental basis for the accuracy of studies on CRC.

### Supplementary Information

Supplementary Information.

## Data Availability

Data generated by this study could be asked from corresponding authors with reasonable request.

## Abbreviations

RT-qPCR: Real-time quantitative polymerase chain reaction
MIQE: Minimum information for publication of RT-qPCR experiments
CRC: Colorectal cancer
HKG: Housekeeping gene
GAPDH: Glyceraldehyde 3-phosphate dehydrogenase
18S: 18s ribosomal RNA
PPIA: Peptidylprolyl isomerase A
ACTB: Actin beta
SDHA: Succinate dehydrogenase complex flavoprotein subunit A
GUSB: Glucuronidase beta
B2M: Beta-2-microglobulin
YWHAZ: Tyrosine 3-monooxygenase/tryptophan 5-monooxygenase activation protein zeta
