# Supplementary material for: Screening and validation of the optimal panel of reference genes in colonic epithelium and relative cancer cell lines
Source: Sci Rep. 2023 Oct 18;13:17777. doi: 10.1038/s41598-023-45174-4 (PMC10584832; doi:10.1038/s41598-023-45174-4)
Supplement: Supplementary file 1 — Supplementary Information. [file 41598_2023_45174_MOESM1_ESM.doc]

**Screening and validation of the optimal panel of reference genes in colonic epithelium and relative cancer cell lines**

Yang Hu^1,#^, Qi Jiang^2,#^, Xiang Zhai^3,#^, Liang Liu^4,*^, Yuntian Hong^3,5,*^

^1^Department of Gastroenterology, The First People's Hospital of Jiande, Hangzhou,311600, China.

^2^Department of Biological Repositories, Zhongnan Hospital of Wuhan University, Wuhan 430071, China.

^3^Key Laboratory of Intestinal and Colorectal Diseases of Hubei Province, Zhongnan Hospital of Wuhan University, Wuhan, 430071, China.

^4^Department of Orthopedic Surgery, Zhongnan Hospital of Wuhan University, Wuhan, 430071, China.

^5^Department of Gastroenterology, Zhongnan Hospital of Wuhan University, Wuhan 430071, China.

**Table S1．The full name, abbreviation, and function of reference genes.**

| **Full name** | **Abbreviation** | **Function** |
| --- | --- | --- |
| Glyceraldehyde-3-phosphate dehydrogenase | *GAPDH* | This gene encodes the key enzyme in glycolysis that widely distributes in various tissues and cells. |
| Actin beta | *ACTB* | This gene encodes one of the cytoskeletal actin. |
| 18s ribosomal RNA | *18S* | This gene encodes small ribosomal subunits that are widely found in animal and plant cells. |
| Peptidylprolyl isomerase A | *PPIA* | This gene encodes a member of the peptidyl-prolyl cis-trans isomerase (PPIase) family. |
| Beta-2-microglobulin | *B2M* | This gene encodes a serum protein found in association with the major histocompatibility complex (MHC) class I heavy chain on the surface of nearly all nucleated cells |
| Succinate dehydrogenase  complex flavoprotein subunit A | *SDHA* | This gene encodes a major catalytic subunit of ubiquinone succinate oxidoreductase that is a component of the respiratory chain. |
| Glucuronidase beta | *GUSB* | This gene encodes a hydrolase that degrades glycosaminoglycans. |
| Tyrosine 3-Monooxygenase+ tryptophan 5-Monooxygenase activation protein zeta | *YWHZA* | This gene encodes the 14-3-3 family of proteins which mediate signal transduction by binding to phosphoserine-containing proteins |

**Table S2．The primers used for a real-time quantitative polymerase chain reaction.**

| **Genes** | **Forward Primer** | **Reverse Primer** | **Annealing(℃)** |
| --- | --- | --- | --- |
| *GAPDH* | GGAGTCCACTGGCGTCTTCA | GTCATGAGTCCTTCCACGATACC | 60 |
| *ACTB* | CCTTCCTGGGCATGGAGTC | TGATCTTCATTGTGCTGGGTG | 58 |
| *18S* | GACTCAACACGGGAAACCTCA | CAGACAAATCGCTCCACCAAC | 59 |
| *PPIA* | TGCTGACTGTGGACAACTCG | TGGAACCCAAAGGGAACTGC | 59 |
| *SDHA* | AAACTCGCTCTTGGACCTGG | TCTTCCCCAGCGTTTGGTTT | 58 |
| *GUSB* | TGCGTAGGGACAAGAACCAC | GGGAGGGGTCCAAGGATTTG | 60 |
| *B2M* | GTTAAGTGGGATCGAGACATGTAAG | TCAAACATGGAGACAGCACTC | 59 |
| *YWHAZ* | GGAGCCCGTAGGTCATCTTG | CGGCAACCTCAGCCAAGTAA | 60 |
| *APC* | GCCAACAAGGCTACGCTATG | TCACACTTCCAACTTCTCGCA | 58 |

*GAPDH*, glyceraldehyde 3-phosphate dehydrogenase; *ACTB*, actin beta; *18S*, 18s ribosomal RNA; *PPIA*, peptidylprolyl isomerase A; *SDHA*, succinate dehydrogenase complex flavoprotein subunit A; *GUSB*, glucuronidase beta; *B2M*, beta-2-microglobulin; *YWHAZ*, Tyrosine 3-Monooxygenase+tryptophan 5-Monooxygenase activation protein zeta; *APC*, APC regulator of WNT signaling pathway.

**Table S3. Paired coefficient of variation (CV) of standardization factor’s variation after introducing a new reference gene analyzed by geNorm in colonic cancer cell lines**

|  | **V2+3** | **V3+4** | **V4+5** | **V5+6** | **V6+7** | **V7+8** |
| --- | --- | --- | --- | --- | --- | --- |
| **HT29** | 0.015 | 0.014171 | 0.012557 | 0.011435 | 0.017566 | 0.016133 |
| **HCT116** | 0.02778 | 0.024222 | 0.030735 | 0.028114 | 0.020298 | 0.023629 |
| **SW480** | 0.025705 | 0.017827 | 0.015042 | 0.017585 | 0.015141 | 0.014491 |
| **SW620** | 0.013823 | 0.015745 | 0.01582 | 0.016711 | 0.016843 | 0.01548 |
| **DLD-1** | 0.024173 | 0.019838 | 0.018792 | 0.015458 | 0.014475 | 0.017029 |
| **LOVO** | 0.011151 | 0.009039 | 0.013589 | 0.011962 | 0.012338 | 0.016245 |
| **RKO** | 0.017217 | 0.022398 | 0.023919 | 0.02151 | 0.018649 | 0.016847 |


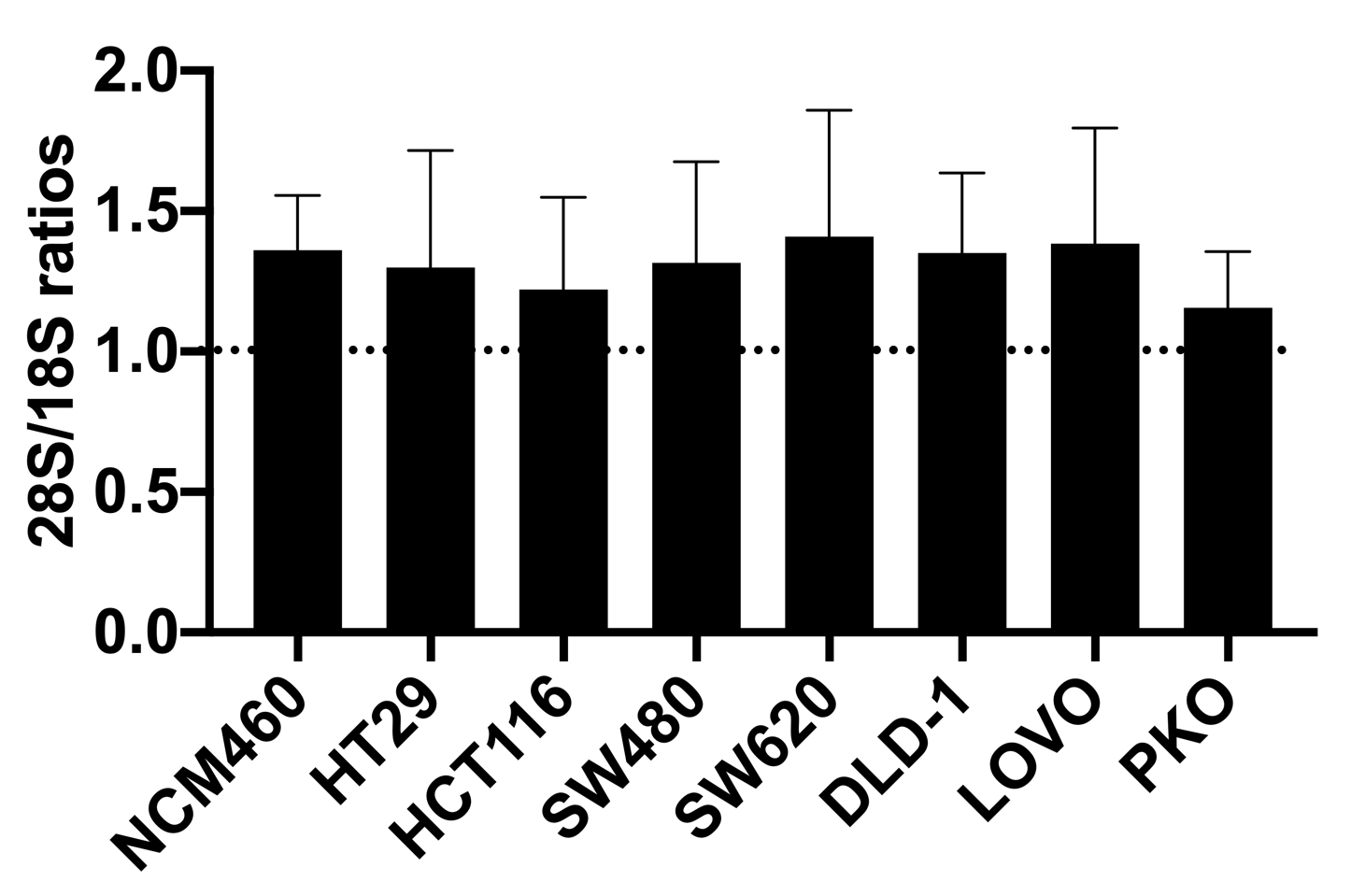


**Fig S1. The 28S and 18S band intensity ratios of RNA gel electrophoresis images.**

**
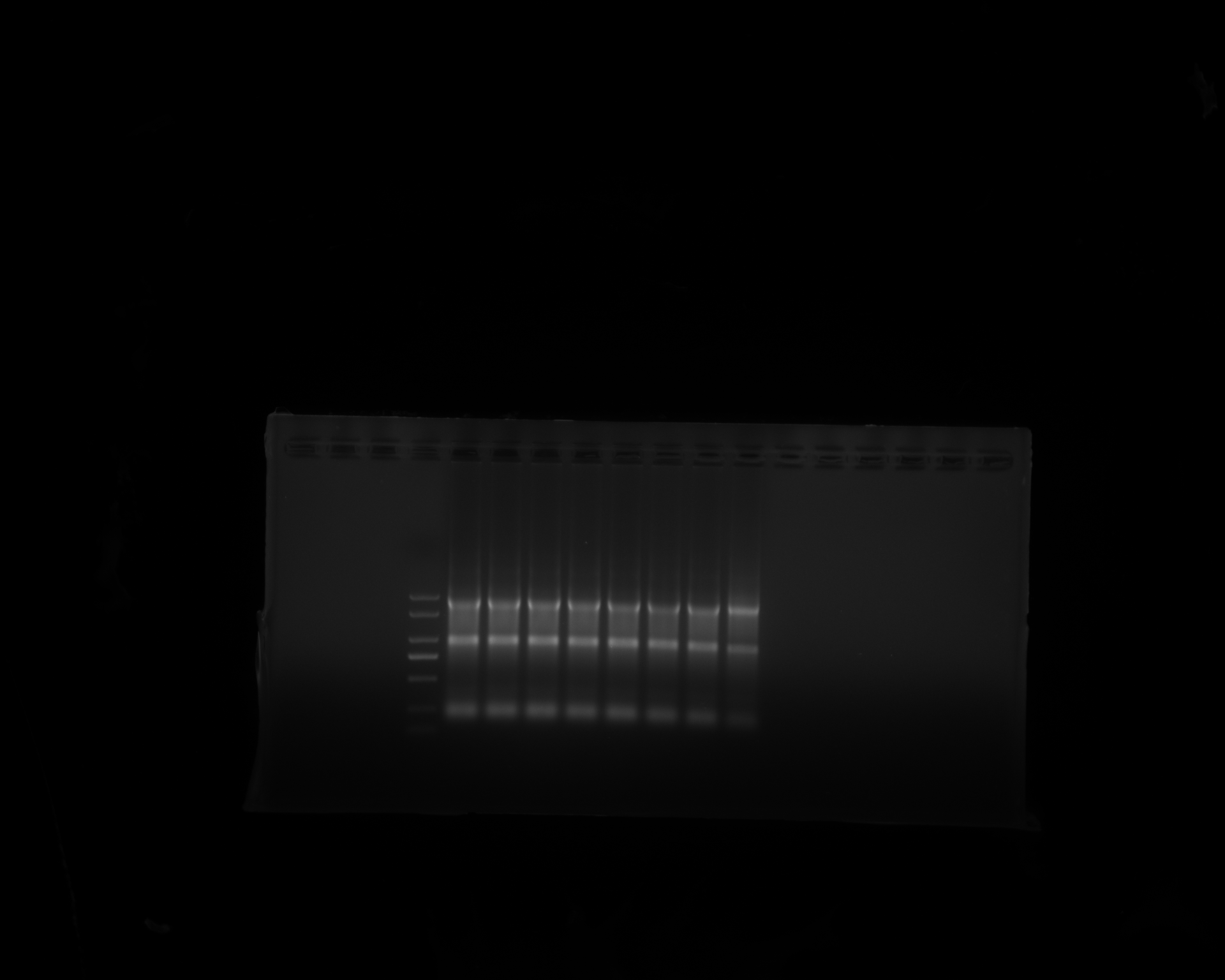
**

**Fig S2. the original, unprocessed gel image in Fig 1A of the manuscript.**
